# Supplementary material for: Off-target effects of CRISPRa on interleukin-6 expression
Source: PLoS One. 2019 Oct 28;14(10):e0224113. doi: 10.1371/journal.pone.0224113 (PMC6816553; doi:10.1371/journal.pone.0224113)
Supplement: S1 Fig — UCSC browser snapshot of conserved regions spanning RP11-326A19.4. Conservation over RP11 exons is restricted to mammals but varies with clades and species. For instance exon 1 is not found in most rodents (Euarchontoglires) but found in more distantly related Afrotheria. Exon 4 shows the highest conservation. The alternate TCONS forms (Human Body Map) reported exhibit less conservation in their last exon (red). (PPTX) [file pone.0224113.s001.pptx]

## Slide 1
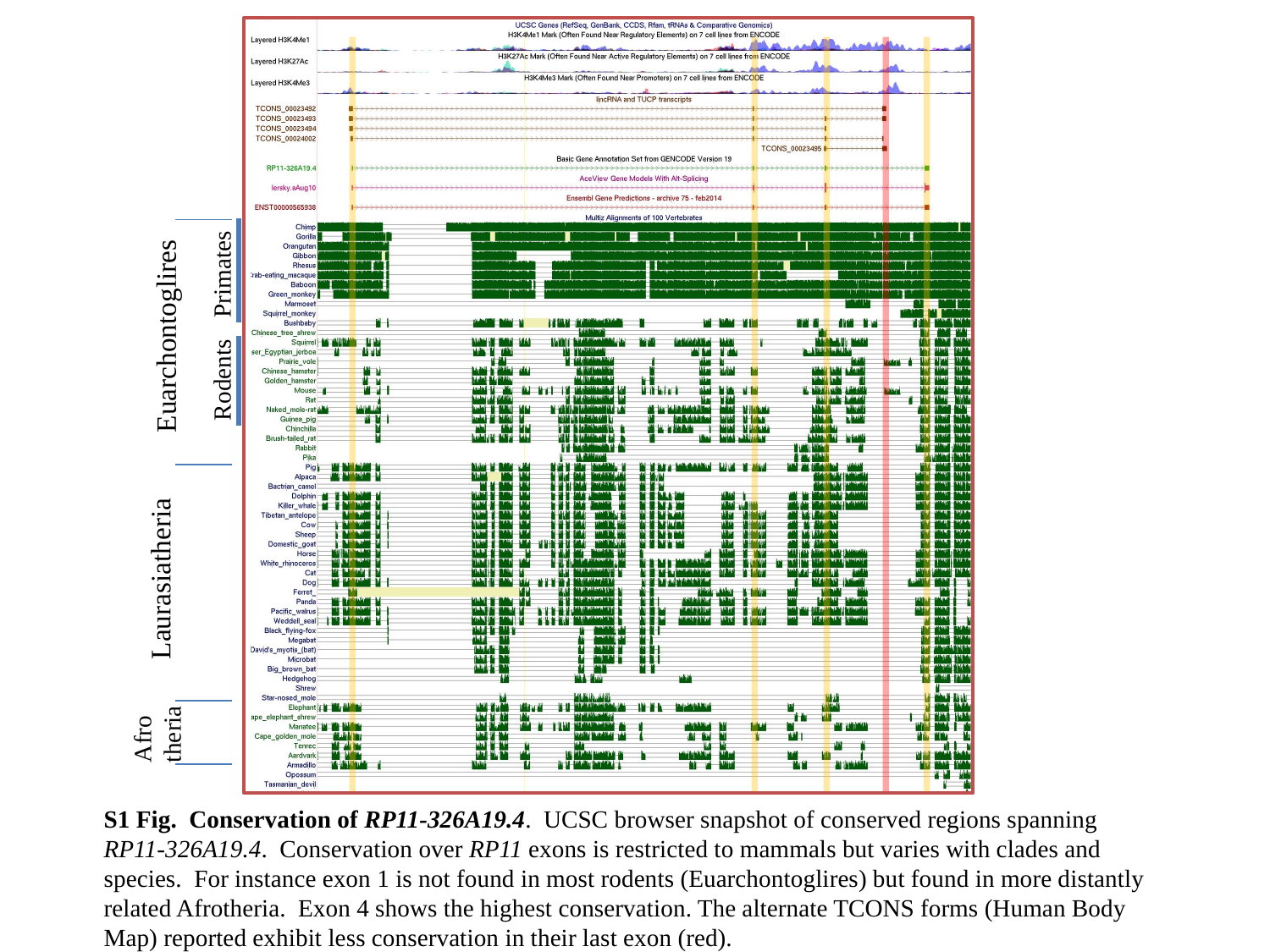

Primates
Euarchontoglires
Rodents
Laurasiatheria
Afro
theria
S1 Fig. Conservation of RP11-326A19.4. UCSC browser snapshot of conserved regions spanning RP11-326A19.4. Conservation over RP11 exons is restricted to mammals but varies with clades and species. For instance exon 1 is not found in most rodents (Euarchontoglires) but found in more distantly related Afrotheria. Exon 4 shows the highest conservation. The alternate TCONS forms (Human Body Map) reported exhibit less conservation in their last exon (red).
