# Supplementary material for: Off-target effects of CRISPRa on interleukin-6 expression
Source: PLoS One. 2019 Oct 28;14(10):e0224113. doi: 10.1371/journal.pone.0224113 (PMC6816553; doi:10.1371/journal.pone.0224113)
Supplement: S2 Fig — Expression levels of genes proximal to RP11 were assessed after RP11 levels were modulated with either VP160 or KRAB in the presence of SG-286. Results are from 4 independent biological replicate (Average ± S.D.). (PPTX) [file pone.0224113.s002.pptx]

## Slide 1
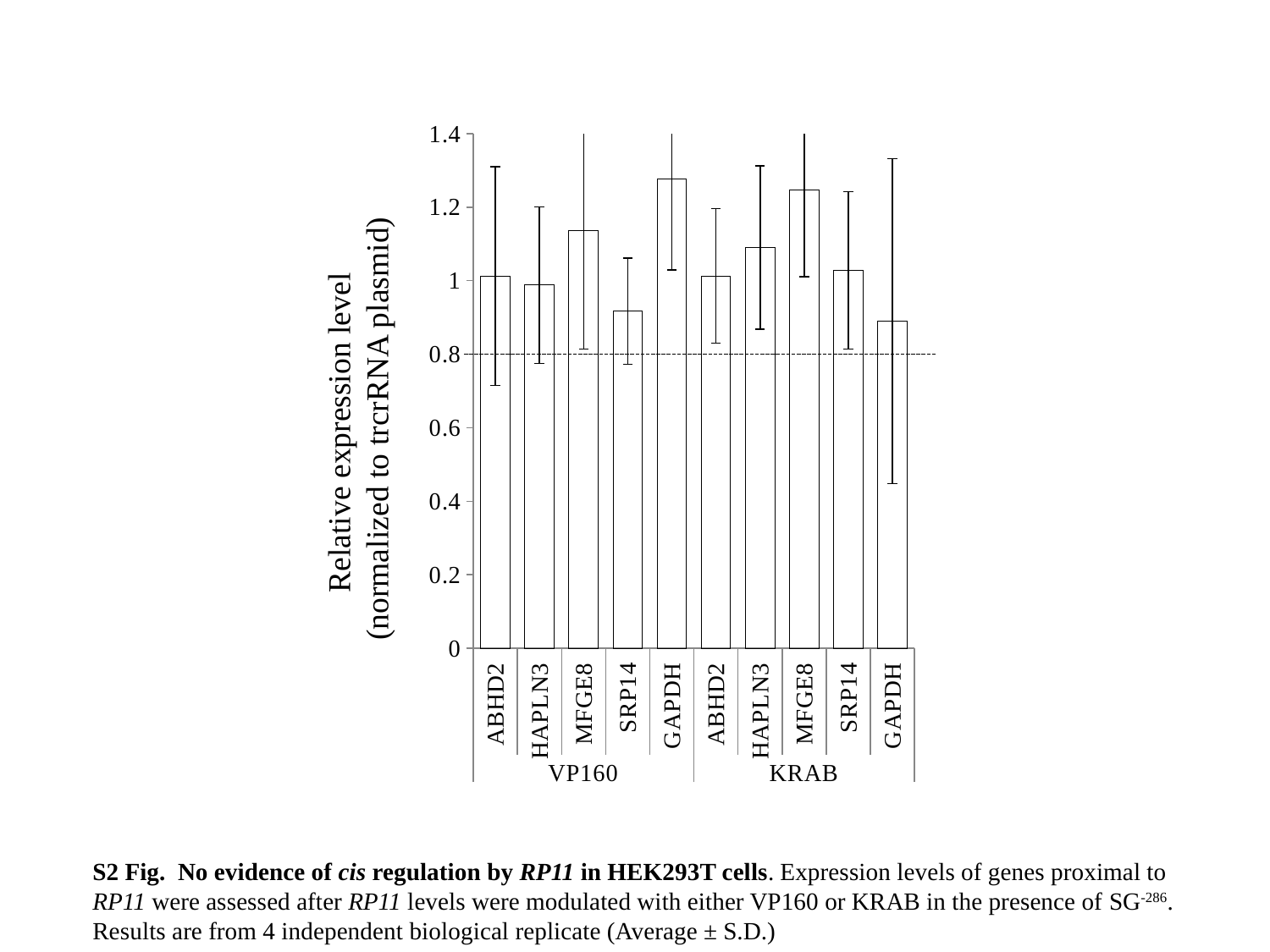

### Chart
| Category | |
|---|---|
| ABHD2 | 1.012580278103832 |
| HAPLN3 | 0.9881613453838275 |
| MFGE8 | 1.1358447302609078 |
| SRP14 | 0.9172259443760452 |
| GAPDH | 1.2776197950017696 |
| ABHD2 | 1.0130344185554214 |
| HAPLN3 | 1.090328114795592 |
| MFGE8 | 1.2458336710741276 |
| SRP14 | 1.0278713056522097 |
| GAPDH | 0.8896792640391042 |Relative expression level
 (normalized to trcrRNA plasmid)
S2 Fig. No evidence of cis regulation by RP11 in HEK293T cells. Expression levels of genes proximal to RP11 were assessed after RP11 levels were modulated with either VP160 or KRAB in the presence of SG-286. Results are from 4 independent biological replicate (Average ± S.D.)
