# Supplementary material for: Off-target effects of CRISPRa on interleukin-6 expression
Source: PLoS One. 2019 Oct 28;14(10):e0224113. doi: 10.1371/journal.pone.0224113 (PMC6816553; doi:10.1371/journal.pone.0224113)
Supplement: S3 Fig — CRISPRa targeting the MFGE8 promoter region in HEK293T cells. Numbers refer to position of sgRNA cognate site relative to start site (RefSeq: NM_001114614). Data represent the average of 3 experiments (± S.D.). (PPTX) [file pone.0224113.s003.pptx]

## Slide 1
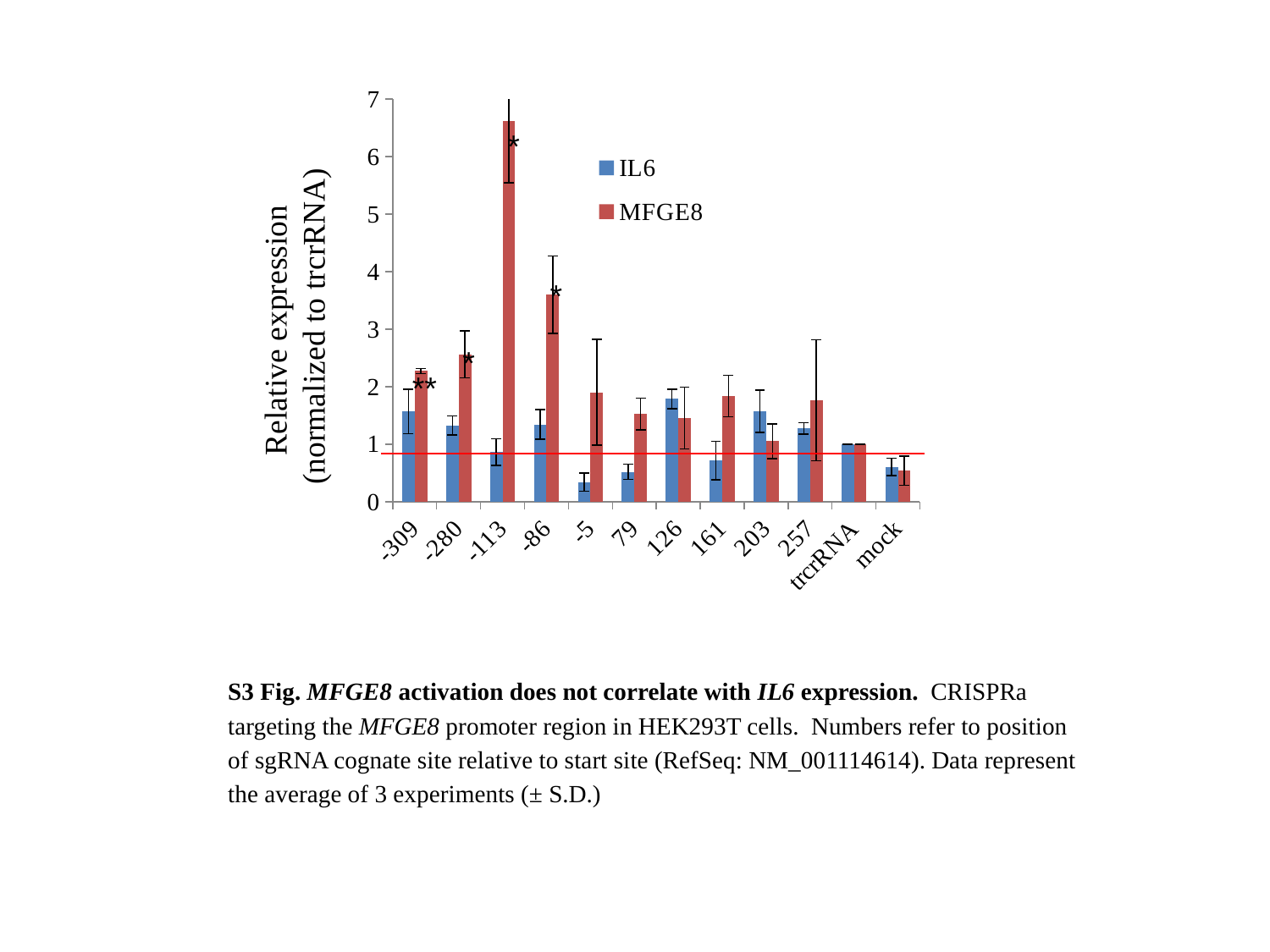

### Chart
| Category | IL6 | MFGE8 |
|---|---|---|
| -309 | 1.5681087064895456 | 2.272072172590365 |
| -280 | 1.324389257702797 | 2.562619803967904 |
| -113 | 0.8650520061446426 | 6.620115409008751 |
| -86 | 1.3421718120154376 | 3.5972356550465974 |
| -5 | 0.34144739721145106 | 1.9024179389511275 |
| 79 | 0.5188051106103363 | 1.523145413618033 |
| 126 | 1.7855197681998947 | 1.4523556572977439 |
| 161 | 0.7144712539447298 | 1.8378060447732834 |
| 203 | 1.5705840455840459 | 1.0506369560918447 |
| 257 | 1.2751424501424502 | 1.7656123249665339 |
| trcrRNA | 1.0 | 1.0 |
| mock | 0.6062411885056382 | 0.5386683986005187 |*
*
Relative expression
(normalized to trcrRNA)
*
**
S3 Fig. MFGE8 activation does not correlate with IL6 expression. CRISPRa targeting the MFGE8 promoter region in HEK293T cells. Numbers refer to position of sgRNA cognate site relative to start site (RefSeq: NM_001114614). Data represent the average of 3 experiments (± S.D.)
