# Supplementary material for: Off-target effects of CRISPRa on interleukin-6 expression
Source: PLoS One. 2019 Oct 28;14(10):e0224113. doi: 10.1371/journal.pone.0224113 (PMC6816553; doi:10.1371/journal.pone.0224113)
Supplement: S4 Fig — Top, CRISPR editing of the SG-505 recognition region. DNA from a putative knock-out clone was PCR amplified, subcloned and individual clones sent for validation by Sanger sequencing. A total of 6 distinct sequences from that clone were obtained, conforming to 2 distinct patterns indicated above. One allele carried point mutations (red) near (-8 to -6) the PAM site while the other allele exhibited a more extensive deletion (DEL). Resulting sequences are expected to be poorly or not recognized by SG-505. Bottom, cells harboring bi-allelic editing of SG-505 binding site were transfected with CRISPRa (VPR) in the presence of either SG-505 or trcrRNA and levels of RP11 and IL6 (and PPIA) were measured 48 h post-transfection. Values are expressed relative to PPIA and then normalized to the corresponding trcrRNA values. Data represent the average values obtained from 3 passages. (PPTX) [file pone.0224113.s004.pptx]

## Slide 1
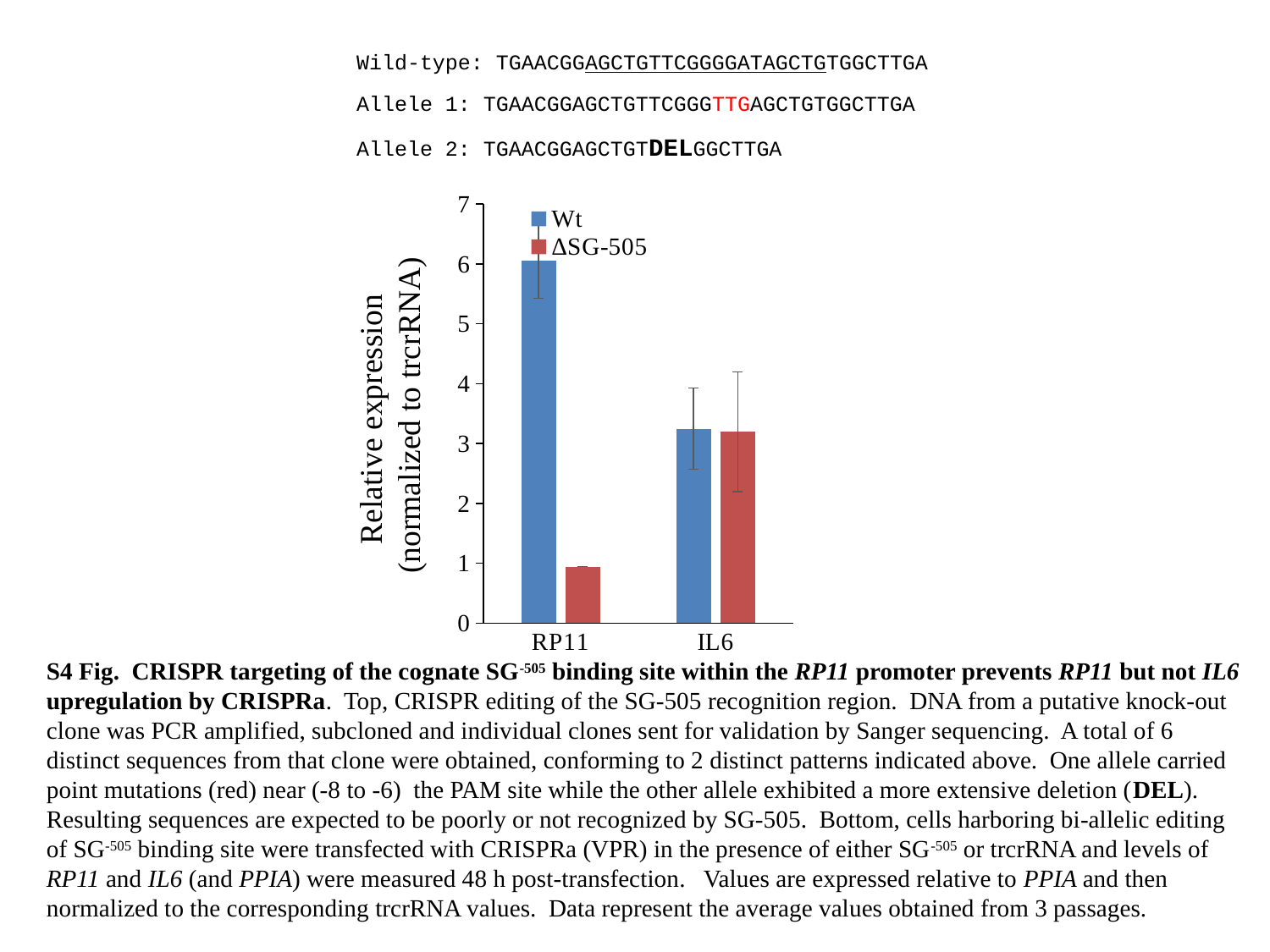

Wild-type: TGAACGGAGCTGTTCGGGGATAGCTGTGGCTTGA
Allele 1: TGAACGGAGCTGTTCGGGTTGAGCTGTGGCTTGA
Allele 2: TGAACGGAGCTGTDELGGCTTGA
### Chart
| Category | Wt | ΔSG-505 |
|---|---|---|
| RP11 | 6.057530469445913 | 0.9421768707482993 |
| IL6 | 3.247502440501176 | 3.195640680931369 |Relative expression
(normalized to trcrRNA)
S4 Fig. CRISPR targeting of the cognate SG-505 binding site within the RP11 promoter prevents RP11 but not IL6 upregulation by CRISPRa. Top, CRISPR editing of the SG-505 recognition region. DNA from a putative knock-out clone was PCR amplified, subcloned and individual clones sent for validation by Sanger sequencing. A total of 6 distinct sequences from that clone were obtained, conforming to 2 distinct patterns indicated above. One allele carried point mutations (red) near (-8 to -6) the PAM site while the other allele exhibited a more extensive deletion (DEL). Resulting sequences are expected to be poorly or not recognized by SG-505. Bottom, cells harboring bi-allelic editing of SG-505 binding site were transfected with CRISPRa (VPR) in the presence of either SG-505 or trcrRNA and levels of RP11 and IL6 (and PPIA) were measured 48 h post-transfection. Values are expressed relative to PPIA and then normalized to the corresponding trcrRNA values. Data represent the average values obtained from 3 passages.
