# Supplementary material for: Off-target effects of CRISPRa on interleukin-6 expression
Source: PLoS One. 2019 Oct 28;14(10):e0224113. doi: 10.1371/journal.pone.0224113 (PMC6816553; doi:10.1371/journal.pone.0224113)
Supplement: S5 Fig — Specificity control for Fig 8; see Fig 8 for additional information. Levels of SRP14 were measured in CRISPRa transfected cells (in the presence of SG-505, SG-286 or trcrRNA) and normalized to the matching vehicle (0.1% DMSO) CRISPRa sample. Data represent the average of 3 experiments (± S.D.). (PPTX) [file pone.0224113.s005.pptx]

## Slide 1
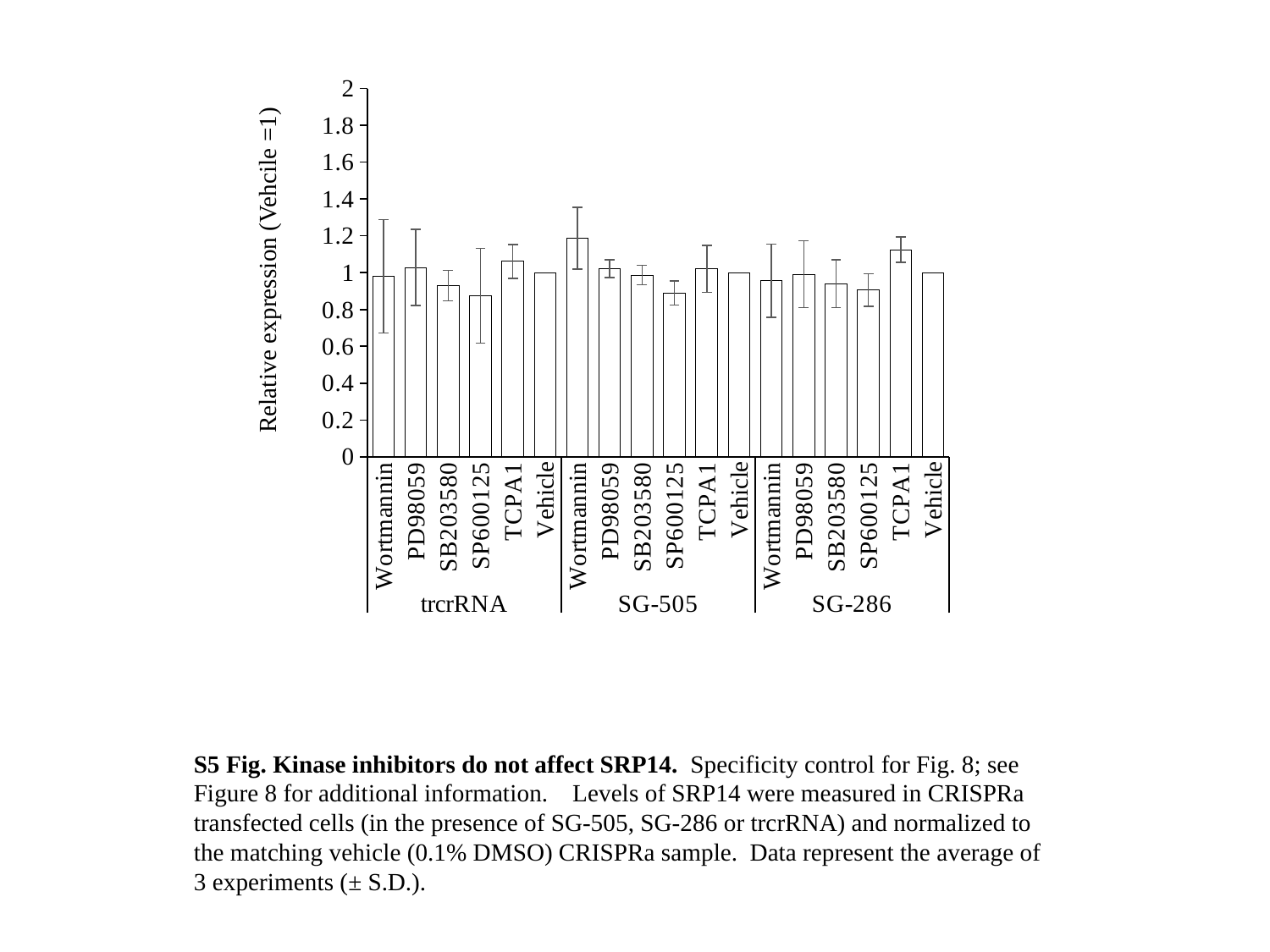

### Chart
| Category | SRP14 |
|---|---|
| Wortmannin | 0.9802613478239057 |
| PD98059 | 1.0285432667082344 |
| SB203580 | 0.9308261881941217 |
| SP600125 | 0.8755270240349017 |
| TCPA1 | 1.0615308880019414 |
| Vehicle | 1.0 |
| Wortmannin | 1.1872962317082418 |
| PD98059 | 1.0213245886723785 |
| SB203580 | 0.9871673364167108 |
| SP600125 | 0.8893775115626659 |
| TCPA1 | 1.0213814542421715 |
| Vehicle | 1.0 |
| Wortmannin | 0.9558933991358254 |
| PD98059 | 0.9914867449750474 |
| SB203580 | 0.9398700533356813 |
| SP600125 | 0.9061964757506612 |
| TCPA1 | 1.124599199215558 |
| Vehicle | 1.0 |Relative expression (Vehcile =1)
S5 Fig. Kinase inhibitors do not affect SRP14. Specificity control for Fig. 8; see Figure 8 for additional information. Levels of SRP14 were measured in CRISPRa transfected cells (in the presence of SG-505, SG-286 or trcrRNA) and normalized to the matching vehicle (0.1% DMSO) CRISPRa sample. Data represent the average of 3 experiments (± S.D.).
