# Supplementary material for: Off-target effects of CRISPRa on interleukin-6 expression
Source: PLoS One. 2019 Oct 28;14(10):e0224113. doi: 10.1371/journal.pone.0224113 (PMC6816553; doi:10.1371/journal.pone.0224113)
Supplement: S6 Fig — Quantification of Western blots (Fig 9). Relative phosphorylation levels (pX/X) were measured and are normalized to the corresponding trcrRNA values (set to 1). Results represent the average of 3 biological replicates (± S.D). *, statistically different (p < 0.05) from trcrRNA. (PPTX) [file pone.0224113.s006.pptx]

## Slide 1
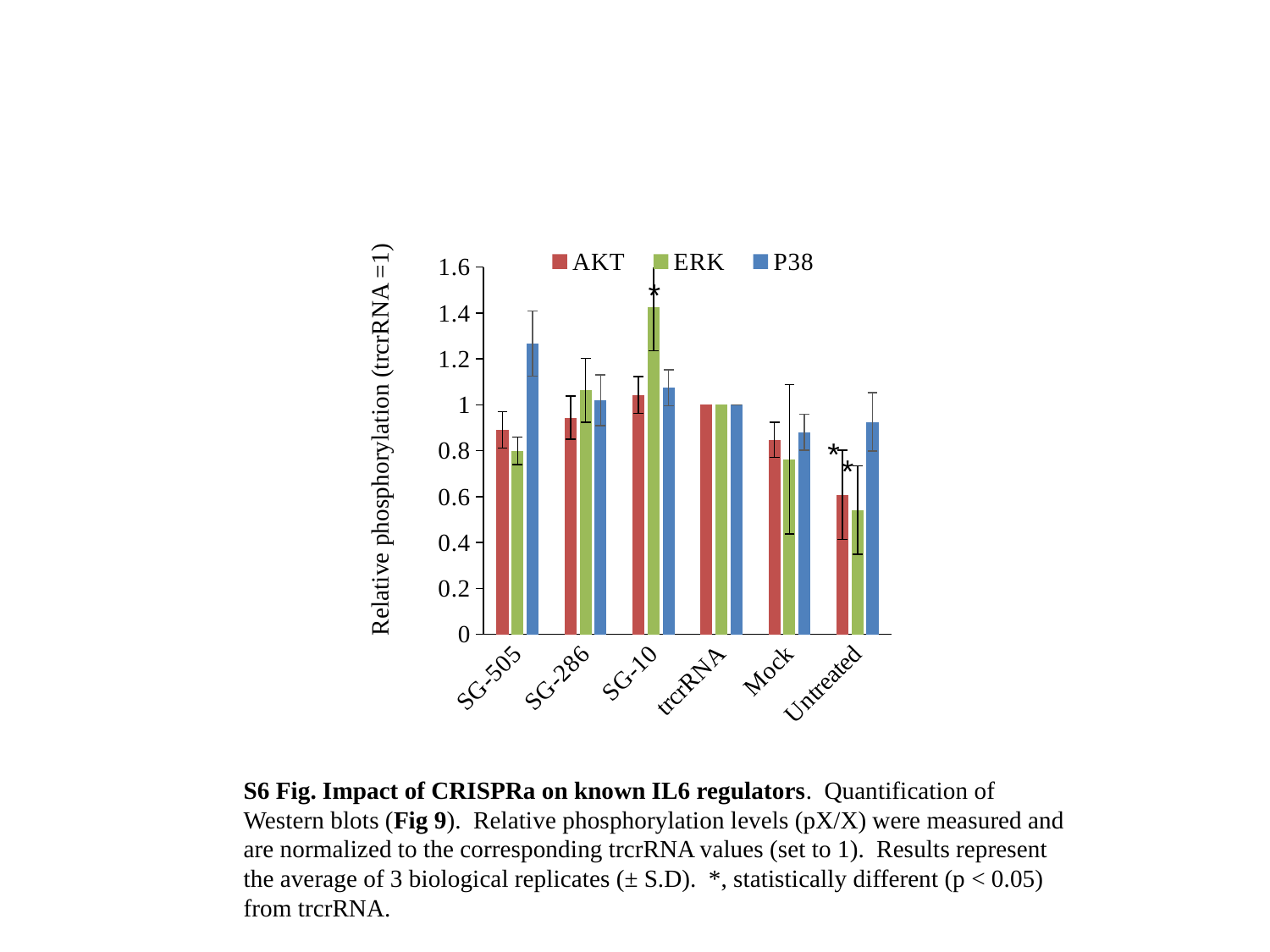

### Chart
| Category | | | |
|---|---|---|---|
| SG-505 | 0.8907171583708976 | 0.7987892856943373 | 1.2659348316044503 |
| SG-286 | 0.9439717477359496 | 1.063053126178162 | 1.0203584168075928 |
| SG-10 | 1.0430062549630872 | 1.4266621245881659 | 1.0738148832259498 |
| trcrRNA | 1.0 | 1.0 | 1.0 |
| Mock | 0.8471176007897631 | 0.7623936713679301 | 0.8809425545992489 |
| Untreated | 0.6080332639775662 | 0.5407565622458882 | 0.9251339503292088 |*
Relative phosphorylation (trcrRNA =1)
*
*
S6 Fig. Impact of CRISPRa on known IL6 regulators. Quantification of Western blots (Fig 9). Relative phosphorylation levels (pX/X) were measured and are normalized to the corresponding trcrRNA values (set to 1). Results represent the average of 3 biological replicates (± S.D). *, statistically different (p < 0.05) from trcrRNA.
