# Supplementary material for: Off-target effects of CRISPRa on interleukin-6 expression
Source: PLoS One. 2019 Oct 28;14(10):e0224113. doi: 10.1371/journal.pone.0224113 (PMC6816553; doi:10.1371/journal.pone.0224113)
Supplement: S7 Fig — THP-1 cells, differentiated to M1 with phorbolesters (100 nM PMA, 72 h), were polarized to M1 with LPS (500 ng/ml) or vehicle (PBS, Ctl) for 2 h, and analyzed by Western blot for the presence of phosphorylated IKKA/B and IKKA. Arrowhead indicates predicted migration position of IKKA/B (84 and 87 kDa, respectively). Approximately 20 μg of THP-1 were loaded per well. HEK293T cells (30 μg, untreated) are included. (PPTX) [file pone.0224113.s007.pptx]

## Slide 1
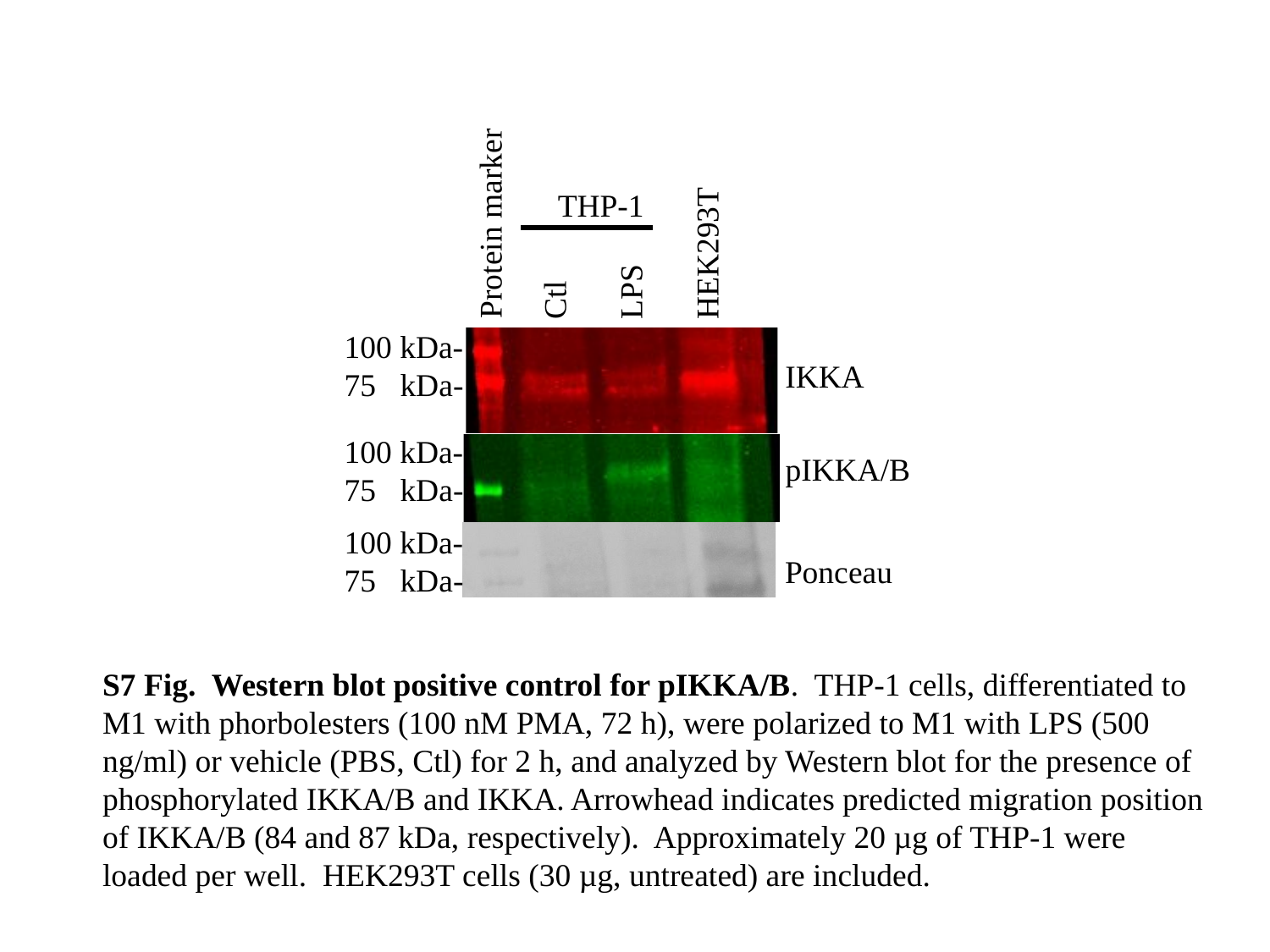

Ctl
LPS
HEK293T
THP-1
Protein marker
100 kDa-
75 kDa-
IKKA
100 kDa-
75 kDa-
pIKKA/B
100 kDa-
75 kDa-
Ponceau
S7 Fig. Western blot positive control for pIKKA/B. THP-1 cells, differentiated to M1 with phorbolesters (100 nM PMA, 72 h), were polarized to M1 with LPS (500 ng/ml) or vehicle (PBS, Ctl) for 2 h, and analyzed by Western blot for the presence of phosphorylated IKKA/B and IKKA. Arrowhead indicates predicted migration position of IKKA/B (84 and 87 kDa, respectively). Approximately 20 µg of THP-1 were loaded per well. HEK293T cells (30 µg, untreated) are included.
