# Supplementary material for: Off-target effects of CRISPRa on interleukin-6 expression
Source: PLoS One. 2019 Oct 28;14(10):e0224113. doi: 10.1371/journal.pone.0224113 (PMC6816553; doi:10.1371/journal.pone.0224113)
Supplement: S8 Fig — Upregulation of RP11 in HuH-7 correlates with increased IL6. HuH-7 cells were transfected with the indicated sgRNA expressing constructs, along with dCAS9-VPR. Values are expressed relative to trcrRNA values, set to 1. * indicates statistically significant (p < 0.05) change with Ctl, as assessed by ANOVA. Data represent the average of 3 experiments (± S.D.). (PPTX) [file pone.0224113.s008.pptx]

## Slide 1
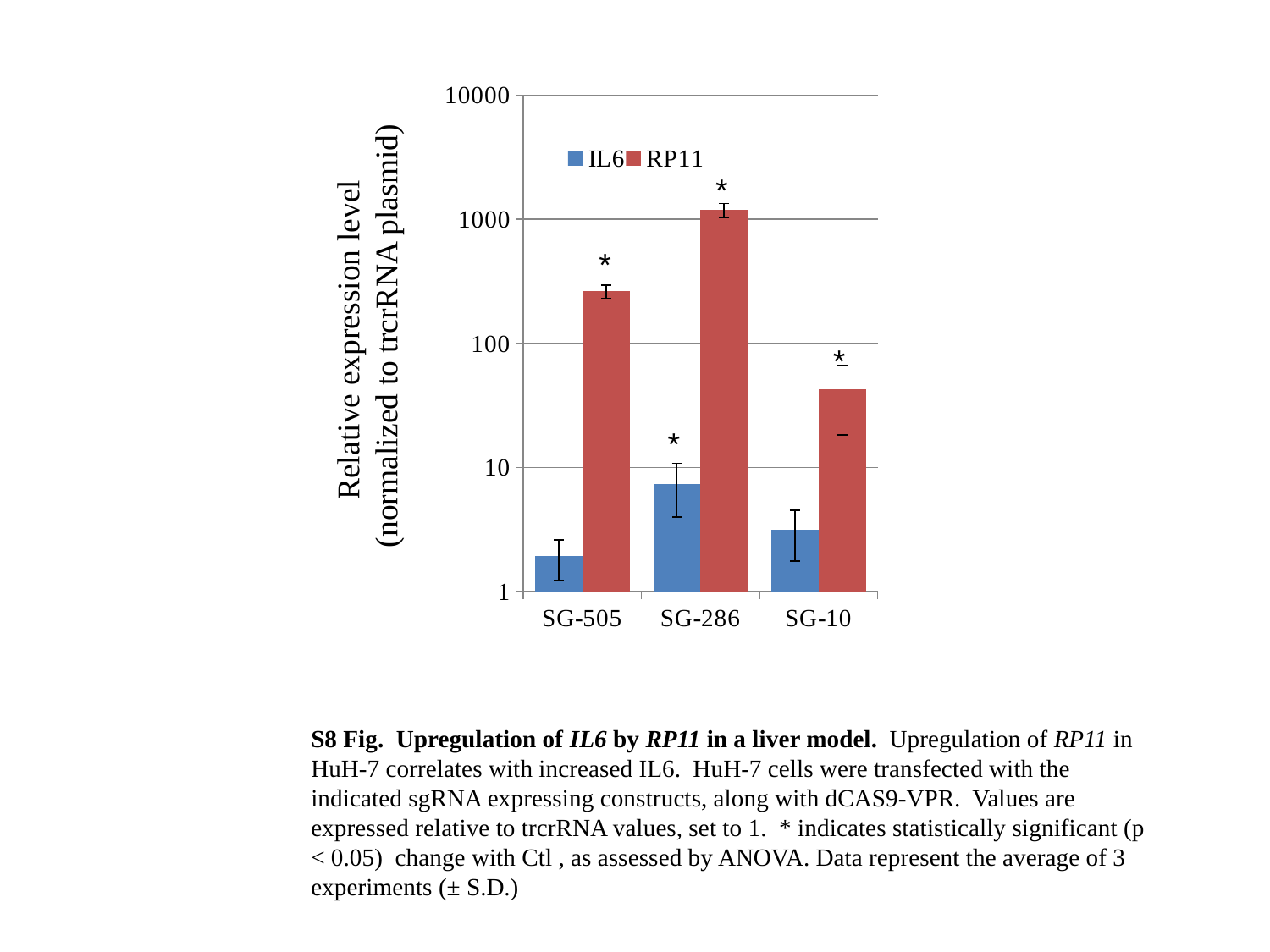

### Chart
| Category | IL6 | RP11 |
|---|---|---|
| SG-505 | 1.9262858072524398 | 263.6257164437142 |
| SG-286 | 7.407160074221821 | 1187.664149074055 |
| SG-10 | 3.147887843961756 | 42.5184322679299 |*
*
Relative expression level
 (normalized to trcrRNA plasmid)
*
*
S8 Fig. Upregulation of IL6 by RP11 in a liver model. Upregulation of RP11 in HuH-7 correlates with increased IL6. HuH-7 cells were transfected with the indicated sgRNA expressing constructs, along with dCAS9-VPR. Values are expressed relative to trcrRNA values, set to 1. * indicates statistically significant (p < 0.05) change with Ctl , as assessed by ANOVA. Data represent the average of 3 experiments (± S.D.)
