# Supplementary material for: Off-target effects of CRISPRa on interleukin-6 expression
Source: PLoS One. 2019 Oct 28;14(10):e0224113. doi: 10.1371/journal.pone.0224113 (PMC6816553; doi:10.1371/journal.pone.0224113)
Supplement: S1 Supplementary Materials — (DOCX) [file pone.0224113.s012.docx]

**qPCR oligos (Forward/Reverse)**

**ABHD2**

TGAGGTCGCCACATCCTTAT

GCAGATGACCATGGTGATATC

**GAPDH**

GATTGGTCGTATTGGG

TCCACGACGTACTCAGC

**HAPLN3**

GCAAAGAGCTGTCCCACCTT

CCTGCACCGCTATGATGTATTC

**IL36B**

CCTCACCACCATCTGATCTAT

TGAATTTCTGCACAGAAGAGAC

**IL6 (Main pair)**

ATCTGGATTCAATGAGGAGACTTG

GGAACTGGATCAGGACTTTTGTACT

**IL6 (Validation pairs)**

Set 1:

GGAGAAGATTCCAAAGATGTAGCC

CTGCCAGTGCCTCTTTGCT

Set 2:

CAGAACGAATTGACAAACAA

CTCATTGAATCCAGATTGGA

**IL6 (pre-mRNA)**

TCGCACTCACTTTTCACTATTCC

ttgaagttggcctcctcatg

**MFGE8**

CGGTGGTTTATGCGAGG

TGTCCAGGCATTGACCATG

**NEAT1**

ggggcggatcggtgttgctt

cccggttccatctgctcgcc

**PPIA**

ACCGTGTTCTTCGACATTGC

TTCTGTGAAAGCAGGAACCC

**RP11-326A19**

Exon 1-2:

GCTGATGCCATGTGGAACAG

CTTCTGCCTGGAGACTAGTAATG

Exon 2-3:

CTGGAACATTACTAGTCTCCAGG

TCTGCTGCAAGCTCACTC

Exon 3-4:

GGATGATTGAATGGATGAATGAACTCC

GAGATAACTCAAAGGACTGAGC

**SRP14**

ACTTCCGGCTCTCACTGCTA

TCAAAGCCCTCCACAGTACC

**Cloning oligos**

Subcloned via MluI site

Region in bold corresponds to promoter region

RP11-326A19.4 promoter cloning

ATATACGCGTT**CTTGACCTTGTGATCCGCCC**

ATATACGCGT**GTCACTGCAGTAGATCCAGAC**

MFGE8 promoter cloning

ATATACGCGT**attattaaaatcacttatttgtaaacagg**

ATATACGCGT**catttttcaccagggaaaa**

**sgRNA oligonucleotides**

All cloned in Bbs1 site of pCRU6

RP11-326A19.4:

-906

caccTTGCCGGGCTGGAGTGCAG

aaacCTGCACTCCAGCCCGGCAA

-567

caccTTGGAAGCAAAGGAATGGA

aaacTCCATTCCTTTGCTTCCAA

-546

caccGAGACAGGACGGCACCCTG

aaacCAGGGTGCCGTCCTGTCTC

-527

caccAGGGAGACGCAGGGCTGAA

aaacTTCAGCCCTGCGTCTCCCT

-505

caccAGCTGTTCGGGGATAGCTG

aaacCAGCTATCCCCGAACAGCT

-334

caccTTCAGACAGACAGACACTT

aaacAAGTGTCTGTCTGTCTGAA

-286

caccGGGAGGAGACTACACACAG

aaacCTGTGTGTAGTCTCCTCCC

-267

caccCCAAGCCCAGAGTCAGTGT

aaacACACTGACTCTGGGCTTGG

-102

caccGTGTAAGGAGGCCAAGAGG

aaacCCTCTTGGCCTCCTTACAC

-10

caccAGCTGAGGCTGCTCCACCG

aaacCGGTGGAGCAGCCTCAGCT

MFGE8:

-309

caccAACAAGGTTAGTGGAGGCTG

aaacCAGCCTCCACTAACCTTGTT

-280

caccGATACTTGCCGATCCGAGAA

aaacTTCTCGGATCGGCAAGTATC

-113

caccCACGACGCGGGACGCCGGTG

aaacCACCGGCGTCCCGCGTCGTG

-86

caccGGCGGAAGAGGCAGATATCG

aaacCGATATCTGCCTCTTCCGCC

-5

caccGGCCAGAGGCGGACTGGGGG

aaacCCCCCAGTCCGCCTCTGGCC

79

caccGAGAAGGCGCCAGAACCCCG

aaacCGGGGTTCTGGCGCCTTCTC

126

caccCGGGGACGCGGGCGCTGGAA

aaacTTCCAGCGCCCGCGTCCCCG

161

caccACAGCGCGGCCAGCAGGCGG

aaacCCGCCTGCTGGCCGCGCTGT

203

caccCCAGGGCGACGAGGAGGCTG

aaacCAGCCTCCTCGTCGCCCTGG

257

caccCCGAGCGGCGCGGGGAGGAG

aaacCTCCTCCCCGCGCCGCTCGG

**Antibodies**

AKT (#4691; Cell Signaling Technology)

AKT pS473 (#9271; Cell Signaling Technology)

ERK(#9107; Cell Signaling Technology)

ERKpT202/Y204 (#4370; Cell Signaling Technology)

IKKA (#AF3768; RandD Systems)

IKKA/BpS176/S180(#2696; Cell Signaling Technology)

P38 (#9212; Cell Signaling Technology)

P38pT180,Y182(#9216; Cell Signaling Technology)
